# Impact of Millet-Based Dietary Intervention on the Nutritional Status of Children in Rural and Tribal Andhra Pradesh, India

**DOI:** 10.7759/cureus.98867

**Published:** 2025-12-10

**Authors:** Arti Gupta, Pulla Sirisha, Joe Amalan, Venkatashiva Reddy B, Rajeev Aravindakshan, Dadi J Madhuri, Durgavajjala P Manaswini, Dokiburra G Rachana, Yusuf N Shaik, Maharshi Deepa

**Affiliations:** 1 Department of Community and Family Medicine, All India Institute of Medical Sciences, Mangalagiri, Mangalagiri, IND; 2 Preventive Medicine, Women Development and Child Welfare Department, Guntur, IND; 3 Nutrition Science, Krishi Vigyan Kendra, Sri Venkateswara Veterinary University, Guntur, IND

**Keywords:** community intervention, millets, preschool children, ragi, undernutrition

## Abstract

Background: According to the International Year of Millets 2023, millets are incredible ancestral, nutrient-rich, climate-resilient grains recognized for their role in preventing malnutrition. However, their consumption among young children remains low due to changing food preferences and limited awareness. This study evaluates the nutritional impact of millet-based interventions among children aged two to six years in rural and tribal regions of Andhra Pradesh, India.

Objectives: To assess millet consumption and nutritional status at baseline, identify socio-demographic determinants of undernutrition and to evaluate the effectiveness of a community-led millet recipe intervention (“Mother’s Kitchen”) on improving dietary intake and growth outcomes.

Methods: A cohort-based survey was conducted among 345 children across Rayalaseema, Uttar Andhra, and Coastal Andhra. Maternal, demographic, and dietary data were collected using structured questionnaires, 24-hour dietary recall (three non-consecutive days), and anthropometric indices using the WHO Anthro software. This involved a 12-week community-led intervention (Mother's Kitchen) involving weekly hands-on millet recipe demonstrations. Data were analyzed using Statistical Product and Service Solutions (SPSS, version 29; IBM SPSS Statistics for Windows, Armonk, NY). A significance level of p < 0.05 was considered statistically significant.

Results: The proportion of children consuming millets increased from 90 (26.2%) to 135 (39.4%). The prevalence of underweight decreased from 120 (35.0%) to 73 (21.3%) (p < 0.001). Children whose mother attended ≥7 sessions had 61% millet consumption, compared with 32.7% for <7 sessions, and a lower prevalence of underweight (RR = 0.673). Socioeconomic status, maternal education, early marriage, breastfeeding practices, and tribal residence were significantly associated with malnutrition.

Conclusion: Millet-based dietary interventions, when delivered through culturally relevant and community-led strategies, significantly improve dietary intake and reduce undernutrition among children. Integrating millets into the Integrated Child Development Services (ICDS) scheme, mid-day meals, and Public Distribution System programs, along with maternal nutrition education, can sustainably enhance child growth outcomes.

## Introduction

Millets, often called nutri-cereals, are small-grained crops valued for their hardiness and role in sustainable agriculture. Public health efforts in India now prioritize reviving traditional foods, with millets at the forefront due to their nutritional and cultural importance [1]. India produces nearly 80% of Asia’s millet supply and 20% of the supply, with Andhra Pradesh having a historically deep connection to these grains. Unlike refined rice and wheat, millets are naturally gluten-free and nutrient-dense, making them a smart dietary choice [2]. They provide B vitamins, calcium, magnesium, zinc, and polyphenols while offering high fiber, low calories, and a low glycaemic index benefits, which support cholesterol reduction and blood sugar control [3]. Varieties such as finger millet (ragi), pearl millet (bajra), foxtail, and little millet are especially rich in iron [4].

Despite these nutritional benefits, millet consumption among young children (two to six years) remains inadequate, and they are particularly vulnerable to nutritional gaps, with iron deficiency anaemia remaining the leading global cause of morbidity and mortality in this age group [5]. Millets help address these deficiencies through high bioavailability of iron, calcium, and other micronutrients. Research shows that including millets, often as ragi sangati, in young children’s diets improves protein and iron intake and supports growth [6].

However, multiple socioeconomic and behavioural barriers impede widespread adoption of millet, contributing to persistent childhood malnutrition [7]. Parental education, especially maternal schooling at the middle or lower levels, also correlates with poor child nutrition [8]. Mothers often cite unfamiliarity, limited availability, and preference for other grains as obstacles [7]. Breastfeeding practices matter too: exclusive breastfeeding protects against underweight, while prelacteal feeding increases risk [9]. Additionally, the rise of fast foods and packaged snacks has reduced millet acceptance among children [10].

Community-based interventions, such as mother-led millet recipe sessions using local and affordable ingredients, have successfully boosted consumption and improved child nutrition [9]. The study aimed to assess millet intake, identify socio-demographic determinants of undernutrition, and evaluate the effectiveness of the "Mother's Kitchen millet" session on dietary intake and anthropometric outcomes.

## Materials and methods

**Figure 1:**
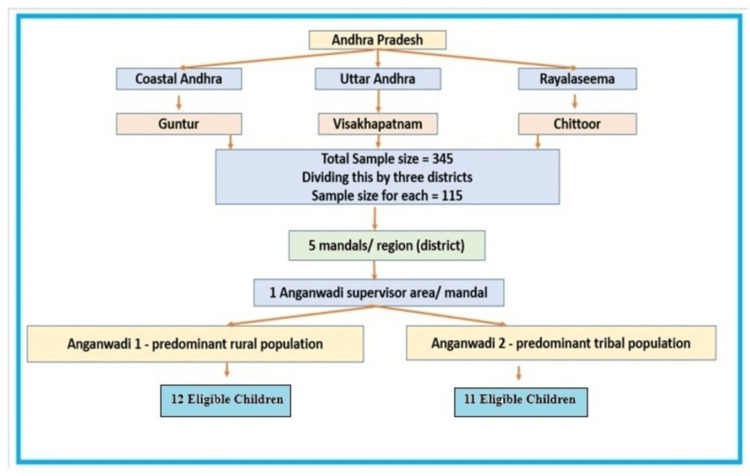
Flow diagram of the study participant recruitment

This is a cohort design study with baseline and end-line comparison conducted in 2024-2025, across three regions of Andhra Pradesh: Guntur district (Uttar Andhra), Visakhapatnam district and Anakapalle district (Coastal Andhra), and Annamayya district (Rayalaseema). The study population was children aged two to six years residing in the study area for ≥6 months, with written informed consent obtained from their parents/caregivers. Acutely sick children were excluded. The sample size calculations assume an estimated incidence with 15% precision and 95% confidence (1.96). A design effect of two and the number of clusters is 10. The cohort included 340 mothers. A total of 115 children were studied in one district. Five mandals were randomly selected in each district using the lottery method. A list of rural and tribal anganwadis in the mandal was received from the Integrated Child Development Services (ICDS) scheme office. Within one mandal, one anganwadi with predominantly rural children and one with predominantly tribal children were randomly selected from the list using a lottery method. A total of 23 children were studied in each mandal. Figure 1 presents the flow diagram of study participant recruitment.

For data collection, trained investigators used structured tools to collect socio-demographic, dietary, anthropometric, and morbidity data from parents/caregivers at baseline and endline (three months apart). For dietary assessment, an interactive, inter-rater-reliable 24-hour recall on three non-consecutive days, using visual aids from dietary software to measure food portions, was conducted. The 24-hour dietary recalls were administered by trained project staff who underwent training conducted both online and in person by experts in dietary assessment. Portion size estimation was facilitated using DietCalc software, supplemented by standardized utensils and photographs to enhance accuracy. Data collection occurred during both the forenoon and afternoon as per the participants' parents/caregivers' availability. Nutrient intake was calculated via the paid version of DietCalc with the Indian Food Composition Table 2017. All children were measured using SECA 210 and 213 instruments, which were calibrated at baseline and endline, respectively; malnutrition was assessed using the World Health Organization (WHO) Anthro (2006 standards) [11]. Anthropometric measurements were taken in duplicate for each child to ensure accuracy and reliability. All measurements were conducted during the forenoon or the afternoon, as per the child's availability. All study tools were piloted in the Center for Rural Health, Primary Health Care Nutakhi, All India Institute of Medical Sciences, Mangalagiri, to refine data collection tools and protocols. Data entry was checked for duplicates, and supervisory checks were conducted once every fortnight for 5% to monitor adherence to protocols.

The Mother's Kitchen program consisted of weekly, hands-on cooking demonstrations conducted over 12 weeks. Sessions were facilitated by a trained nutritionist, with step-by-step, quantity-wise recipe preparation, recipe samples, and community health workers at anganwadi centers, or participants homed in groups of 5-10 mothers. Each week featured a demonstration of two to three age-appropriate, culturally acceptable millet recipes using locally available ingredients. The intervention covered nine true millets (finger millet/ragi, pearl millet/bajra, foxtail millet, little millet, kodo millet, proso millet, barnyard millet, brown top millet, and sorghum/jowar) and two pseudo cereals (amaranth and buckwheat). Recipes were explicitly designed for children aged two to six years. Emphasizing palatability, ease of preparation, and nutrient density. Mothers were shown visual diaries with a step-by-step and quantity-wise recipe preparation, along with samples of recipes.

The Institute Ethics Committee of All India Institute of Medical Sciences, Mangalagiri, approved the study. All the mothers were given information on the research and provided a patient information sheet. The mothers who accepted to participate in the study were asked to provide written informed consent. There are no incentives/compensation provided to mothers.

All 345 mothers were being followed up as per protocol. Two mothers were relocated from the study area, resulting in attrition and leaving 343 mothers for the end-line analysis. Data were entered in Microsoft Excel (Microsoft® Corp., Redmond, WA). It was analysed using Statistical Product and Service Solutions (SPSS, version 29; IBM SPSS Statistics for Windows, Armonk, NY), WHO Anthro, and DietCalc. Descriptive statistics were reported; associations were tested via the chi-square test or Fisher’s exact test. Exposure was operationally defined as attending seven or more sessions of the Mother Kitchen Millet talk. Effectiveness assessed using Relative Risk and logistic regression (bivariate then multivariate, p<0.2 entry, p<0.05 significance).

## Results

A total of 345 children (180 rural, 165 tribal) were enrolled across three regions. Two children were lost to follow-up, yielding an attrition rate of 0.6%.

Table 1 shows the age distribution of the studied children: in Rayalaseema, 48-60 months: rural 20 (33.3%) and tribal 17 (30.9%); and in Uttar Andhra, 36-47 months: tribal 31 (56.4%); 24-35 months: rural 19 (31.7%) (Table 1).

**Table 1:** Distribution of the studied children by age (n=345)

Child's age in months	Rayalaseema				Uttar Andhra				Coastal Andhra				Total			
	Rural		Tribal		Rural		Tribal		Rural		Tribal		Rural		Tribal	
Category	n	%	n	%	n	%	n	%	n	%	n	%	n	%	n	%
24-35	14	23.33	14	25.45	19	31.7	11	20	5	8.33	3	5.45	38	21.11	28	16.97
36-47	15	25	12	21.82	14	23.3	31	56.36	21	35	20	36.36	50	27.78	63	38.18
48-60	20	33.33	17	30.91	17	28.3	11	20	26	43.33	15	27.27	63	35	43	26.06
61-71	11	18.33	12	21.82	10	16.7	2	3.64	8	13.33	17	30.91	29	16.11	31	18.79
Total	60	100	55	100	60	100	55	100	60	100	55	100	180	100	165	100
Fischer's Exact/Chi-Square Test	0.403				14.986				6.511				6.211			
Degrees of freedom (df)	3				3				3				3			
p value	0.94				0.002				0.089				0.102			

Table 2 shows gender distribution: (1) Rayalaseema - rural: 31 (51.7%) female and 29 (48.3%) male; tribal: 27 (49.1%) female and 28 (50.9%) male; (2) Uttar Andhra - rural: 33 (55.0%) female and 27 (45.0%) male; tribal: 30 (54.5%) female and 25 (45.5%) male; and (3) Coastal Andhra - rural: 36 (60.0%) male and 24 (40.0%) female; tribal: 31 (56.4%) female and 24 (43.6%) male.

**Table 2:** Distribution of the studied children by gender (n=345)

Gender	Rayalaseema				Uttar Andhra				Coastal Andhra				Total			
	Rural		Tribal		Rural		Tribal		Rural		Tribal		Rural		Tribal	
Category	n	%	n	%	n	%	n	%	n	%	n	%	n	%	n	%
Female	31	51.67	27	49.09	33	55	30	54.55	24	40	31	56.36	88	48.89	88	53.33
Male	29	48.33	28	50.91	27	45	25	45.45	36	60	24	43.64	92	51.11	77	46.67
Total	60	100	55	100	60	100	55	100	60	100	55	100	180	100	165	100
Fischer's Exact/Chi-Square Test	0.76				0.002				3.079				0.68			
Degrees of freedom (df)	1				1				1				1			
p value	0.783				0.961				0.079				0.409			

Table 3 shows the median daily intake increased from 36.67 g (IQR: 50) at baseline to 50 g (IQR: 16.67) at endline across all sites. In Rayalaseema, rural millet intake rose from 38.34 g (IQR: 41.67) to 50 g (IQR: 8.34), while tribal median remained at 50 g. Uttar Andhra saw rural millet intake slightly drop from 30 g to 28.34 g, but tribal millet intake rose from 3.33 g to 13.33 g. Coastal Andhra tribal millet intake increased from 13.33 g to 16.67 g. 

**Table 3:** Amount of millet consumed by the studied child using 24-hour dietary recall (n=345) at baseline and endline

District	Site	Baseline			Endline		
		n	Median (g/day)	Interquartile Range (IQR) (g/day)	n	Median (g/day)	Interquartile Range (IQR) (g/day)
Rayalaseema	Rural	38	38.34	41.67	58	50.00	8.34
	Tribal	41	50.00	58.34	47	50.00	0.00
	Total	79	43.33	60	105	50.00	8.34
Uttar Andhra	Rural	1	30.00	-	16	28.34	38.83
	Tribal	1	3.33	-	7	13.33	20
	Total	2	16.67	-	23	26.67	36.66
Coastal Andhra	Tribal	9	13.33	10.84	7	16.67	3.33
	Total	9	13.33	10.84	7	16.67	3.33
Total	Rural	39	36.67	40	74	50.00	16.67
	Tribal	51	36.67	60	61	50.00	20
	Total	90	36.67	50	135	50.00	16.67

Table 4 shows baseline and endline millet recipes: Ragi Malt increased from 7 (7.87%) to 12 (8.22%) and Ragi Dosa rose from 1 (1.12%) to 10 (6.85%). New recipes appeared at the endline: Jowar Dosa (4, 2.74%), Ragi Idli (5, 3.42%), Ragi Laddu (3, 2.05%), Jowar Roti (6, 4.11%), and Jowar Popcorn (105, 71.92%).

**Table 4:** Pattern of millet intake among the studied children at baseline and endline using 24-hour dietary recall *Multiple options

Millet recipe	Baseline		Endline	
	n	%	n	%
Ragi Malt	7	7.87	12	8.22
Ragi Dosa	1	1.12	10	6.85
Ragi Sangati	81	91.01	1	0.68
Jowar Dosa	-	-	4	2.74
Ragi Idli	-	-	5	3.42
Ragi Laddu	-	-	3	2.05
Jowar Roti	-	-	6	4.11
Jowar Popcorn	-	-	105	71.92
Total	89	100	146	100

Table 5 shows endline millet consumption in children rose significantly from 90 (26.2%) at baseline to 135 (39.4%) (Z = -5.267, p < 0.001). Children of mothers attending ≥7 recipe talks had 71 (61.0%) consumption vs. 74 (32.7%) for p-value < 0.001.

**Table 5:** Comparison of millet intake diet measured using 24-hour dietary recall among children at the endline survey and local millet recipe talk *reference; # Baseline comparative; ^ Statistically significant

Variable	Proportion of children who consumed millets							
	n	Baseline survey #		Endline survey		Relative Risk (95%CI)	Z statistics	P value
		n	% (95%CI)	n	% (95%CI)			
Step 1: Excluding attrition								
Number of children excluding attrition	343	90	26.2(21.9 to 31.1)	135.0	39.4(34.3 to 44.6)	-	-5.267	<0.001^
Step 2: Stratifying mothers with respect to the number of local millet recipe talks attended								
>=7*	117	32		61.0		2.23 (1.42 to 3.53) ^	-4.849	<0.001^
<7	226	58	25.7 (20.4-31.7)	74.0	32.7 (27.0-39.1)		-2.530	0.006^

Table 6 shows that Uttar Andhra had the highest severe underweight rate (19, 34.6%) in tribal children, while Rayalaseema had the lowest overall underweight prevalence; tribal areas showed significant variance in underweight categories (p = 0.041).

**Table 6:** Proportion of underweight with severity among the studied children at baseline (n=345)

Underweight among children	Rayalaseema				Uttar Andhra				Coastal Andhra				Total			
	Rural		Tribal		Rural		Tribal		Rural		Tribal		Rural		Tribal	
Category	n	%	n	%	n	%	n	%	n	%	N	%	n	%	n	%
Moderate	5	8.33	8	14.55	14	23.3	12	21.82	11	18.33	18	32.73	30	16.67	38	23.03
Severe	4	6.67	2	3.64	11	18.3	19	34.55	7	11.67	10	18.18	22	12.22	31	18.79
Normal	51	85.00	45	81.82	35	58.3	24	43.64	42	70.00	27	49.09	128	71.11	96	58.18
Total	60	100.00	55	100.00	60	100.0	55	100.00	60	100.00	55	100.00	180	100.00	165	100.00
Fisher's exact/ Chi square	1.519				4.128				5.273				6			
Degrees of freedom (df)	2				2				2				2			
p value	0.468				0.127				0.072				0.041			

Table 7 shows baseline underweight risk factors in 345 children (121, 35%). Maternal factors are as follows: age >25 years (68, 56.2%), early marriage (25, 20.7%), non-working (80, 66.1%), low education (54, 44.6%), low paternal education (58, 47.9%), low socioeconomic status (104, 86%), consanguinity (37, 30.6%), and low standard of living index (35, 28.9%). Child factors are follows: female (62, 51.2%), age 49-72 months (71, 58.7%), pre-lacteal feeding (45, 37.2%), exclusive breastfeeding (55, 45.5%), full immunization (101, 83.5%), and low millet intake (14, 11.6%).

**Table 7:** Unadjusted and adjusted relative risk for variables, risk factors for child baseline underweight (n=345)

Variable	Category	Present (n=121)		Absent (n=224)		Total (n=345)		Unadjusted Odds ratio	OR CI 95%		p value	Adjusted OR	AOR CI 95%		p value
		n	%	n	%	n	%		Upper	lower			upper	lower	
Mother age (years)	<=25 (ref)	53	43.8	107	47.8	160	46.4	0.0852	0.546	1.329	0.481	-	-	-	-
	>25	68	56.2	117	52.2	185	53.6								
Mother's age at marriage (years)	<18 (ref)	25	20.7	30	13.4	55	15.9	1.684	0.939	3.021	0.078	-0.425	0.264	-1.115	0.227
	>=18	96	79.3	194	86.6	290	84.1								
Mother occupation	Not working (ref)	80	66.1	174	77.7	254	73.6	0.561	0.343	0.916	0.02	0.331	0.938	-0.276	0.286
	Working	41	33.9	50	22.3	91	26.4								
Father occupation	labour (ref)	76	62.8	140	62.5	216	62.6	1.013	0.641	1.601	0.955	-	-	-	-
	Others	45	37.2	84	37.5	129	37.4								
Mother's education status	Middle school or less (ref)	54	44.6	62	27.7	116	33.6	2.106	1.325	3.345	0.001	-0.288	0.328	-0.904	0.36
	More than Middle school	67	55.4	162	72.3	229	66.4								
Father's education status	Middle school or less (ref)	58	47.9	80	35.7	138	40.0	1.657	1.057	2.597	0.027	-0.072	0.523	-0.666	0.813
Socioeconomic status	More than Middle school	63	52.1	144	64.3	207	60.0								
	Middle class or lower (ref)	104	86.0	173	77.2	277	80.3	1.803	0.989	3.287	0.0052	-0.863	-0.17	-1.556	0.015
	More than the middle class	17	14.0	51	22.8	68	19.7								
Consanguineous marriage of parents	No (ref)	84	69.4	162	72.3	246	71.3	0.869	0.535	1.411	0.57	-	-	-	-
	Yes	37	30.6	62	27.7	99	28.7								
Standard of living index	low (ref)	35	28.9	38	17.0	73	21.2	1.992	1.178	3.369	0.009	0.075	0.782	-0.631	0.835
	Medium or more	86	71.1	186	83.0	272	78.8								
Child gender	Male (ref)	59	48.8	110	49.1	169	49.0	0.986	0.634	1.535	0.951	-	-	-	-
	Female	62	51.2	114	50.9	176	51.0								
Child's age in months	24-48	50	41.3	129	57.6	179	51.9	0.519	0.331	0.812	0.004	0.877	1.419	0.336	0.002
	49-72	71	58.7	95	42.4	166	48.1								
Child known case of disease	yes (ref)	7	5.8	10	4.5	17	4.9	0.761	0.282	2.053	0.589	-	-	-	-
	No	114	94.2	214	95.5	328	95.1								
Prelacteal	Yes	45	37.2	60	26.8	105	30.4	0.618	0.385	0.991	0.045	0.236	0.861	-0.388	0.458
	No (ref)	76	62.8	164	73.2	240	69.6								
Exclusive breastfeeding	Yes	55	45.5	69	30.8	124	35.9	1.872	1.186	2.955	0.007	-0.492	0.123	-1.107	0.117
	No (ref)	66	54.5	155	69.2	221	64.1								
Child immunized for age	Fully (ref)	101	83.5	124	55.4	225	65.2	4.073	2.356	7.04	<0.001	-1.093	-0.304	-1.883	0.007
	Partially	20	16.5	100	44.6	120	34.8								
Child millet consumed in 24-hour dietary recall	Yes	14	11.6	76	33.9	90	26.1	0.255	0.137	0.475	<0.001	1.362	2.289	0.436	0.004
	No	107	88.4	148	66.1	255	73.9								
Site	Rural	52	43.0	128	57.1	180	52.2	0.565	0.884	0.362	0.012	0.649	1.209	0.088	0.023
	Tribal	69	57.0	96	42.9	165	47.8								

Table 8 shows that, among 343 children, underweight prevalence decreased from 120 (35.0%) at baseline to 73 (21.3%) at endline (p < 0.001). In the ≥7 recipe talks group, underweight decreased from 34 (29.1%) to 20 (17.1%) (RR = 0.673, p = 0.002).

**Table 8:** Comparison of underweight among the children at the endline survey and the local millet recipe talk *reference; # Baseline comparative; ^ Statistically significant

Variable	Underweight child							
	N	Baseline survey #		Endline survey		Relative Risk (95%CI)	Z statistics	P value
		n	% (95%CI)	N	% (95%CI)			
Step 1: Excluding attrition								
Number of children excluding attrition	343	120	35.0 (30.1-40.2)	73.0	21.3 (17.3-25.9)	-	5.039	<0.001
Step 2: Stratifying mothers with respect to the number of local millet recipe talks attended								
>=7*	117	34	29.1 (21.6-37.9)	20.0	17.1 (11.3-25.0)	0.673 (0.380-1.192)	2.858	0.002
<7	226	86	38.1(32.0 -44.5)	53.0	23.5 (18.3-29.4)		4.158	<0.001

## Discussion

The present study provides community-level evidence on the role of millets in improving nutritional outcomes among children aged two to six years in rural and tribal Andhra Pradesh. The demographic profile showed a balanced age distribution, with a higher concentration in the 36-60-month age group, consistent with declining fertility rates and effective family planning in rural India [10]. Gender ratios were near parity across regions, with minor male predominance in Coastal Andhra, aligning with National Family and Health Survey (NFHS)-5 trends of gradual normalization in the sex ratio at birth [12].

Morbidity was predominantly acute, with over 90% of children free from chronic conditions. Respiratory symptoms were the most reported, underscoring the persistent burden of acute respiratory infections in rural under-five populations [13]. The low detection of chronic illnesses likely reflects underdiagnosis in community settings rather than actual absence, emphasizing the need for enhanced paediatric screening [14].

Feeding practices varied significantly by region. Exclusive breastfeeding rates were high in Rayalaseema and Uttar Andhra (80-92%) but alarmingly low in Coastal Andhra (5-25%), particularly among tribal mothers, where top feeding predominated. This disparity mirrors NFHS-5 findings and is attributable to maternal employment, urbanization, and access to commercial milk substitutes [14-16]. Prelacteal feeding remained culturally entrenched in Uttar Andhra (56-63%), despite awareness campaigns, highlighting the influence of social norms [16]. Immunization coverage, however, was exemplary (92-100%), exceeding national averages and reflecting the success of initiatives such as Mission Indradhanush [12,17].

Maternal profiles revealed that young caregivers (21-30 years) were the primary decision-makers for child feeding [18,19]. Early marriage persisted in tribal communities (18-33%), exceeding national averages [20], and was associated with reduced dietary autonomy. Educational disparities were stark, with one in five tribal mothers being illiterate, a known determinant of poor dietary diversity. Socioeconomic constraints further shaped dietary patterns: tribal households relied heavily on daily wage labour and clustered in the lowest income quintile, sustaining millet consumption due to affordability and cultural continuity [20,21]. In contrast, upwardly mobile rural families increasingly favoured refined cereals, reflecting aspirational dietary shifts [22].

Millet consumption among children was dominated by ragi sangati, with limited variety or preparation options. This pattern aligns with traditional practices in South India, where ragi-based complementary foods are valued for digestibility and micronutrient density [3,18]. However, low-frequency and variety underscore barriers in acceptability, preparation complexity, and caregiver knowledge [8,23]. The 12-week Mother’s Kitchen intervention, with weekly hands-on millet recipe demonstrations, significantly improved dietary intake and nutritional status. A marked decline in underweight (from 35.0% to 21.3%, p < 0.001) was found. Children whose mothers attended ≥7 sessions showed superior outcomes (RR = 0.673 for underweight reduction), highlighting dose-response efficacy.

These gains are mechanistically supported by millet’s nutrient profile: high-quality protein (8-12 g/100 g), exceptional calcium in finger millet (300-350 mg/100 g), and iron (2-8 mg/100 g) with enhanced bioavailability through traditional processing such as fermentation and germination [24,25]. Comparative studies consistently favour millet-based over rice-based diets for anthropometric recovery, haemoglobin improvement, and sustained growth velocity [3,26,27]. Biofortified varieties further amplify iron and zinc absorption, with cognitive benefits observed in young children [27].

The intervention’s success hinges on maternal engagement and child-centric recipe design. Integration of millets with familiar foods (e.g., milk, pulses) improved palatability and compliance, outperforming direct supplementation models [3]. Community-based cooking demonstrations empowered mothers as agents of change, aligning with evidence that family-centered nutrition education yields higher adherence than institutional programs [28].

A key strength of this study lies in its rigorous cohort design with baseline and endline comparisons over a defined period across diverse regions of Andhra Pradesh. The use of cluster selection increased generalisability, while the training and quality control measures ensured data integrity. The limitations of this study included the 24-hour dietary recall obtained from the mother; the cohort inference study needs to be further validated by a larger randomized study; and the absence of a control group significantly limits the ability to draw causal inferences.

Despite high awareness of millet’s nutritional value, persistent barriers continue to limit widespread adoption. Taste preferences, especially among children, pose a significant challenge, often favoring Westernized or processed foods over traditional millet dishes. Culinary knowledge gaps and limited cooking confidence further limit the use of regular millet, while accessibility and affordability issues, particularly for less common millet varieties, compound the problem. To translate these findings into practice, we recommend scaling the Mother’s Kitchen model statewide through Anganwadi and self-help groups using child-friendly millet recipes, millet inclusion in ICDS, mid-day meals, and Pradhan Mantri Poshan Shakti Nirman (PM POSHAN) with biofortified varieties, and training primary healthcare workers in millet counselling paired with point-of-care anaemia screening. Targeted literacy-nutrition programs for mothers to enhance dietary autonomy. The subsidized Public Distribution System should strengthen millet supply chains.

## Conclusions

This study confirms millets’ intake among Indian children, identifies socio-cultural and economic barriers to their growth, and demonstrates the success of the weekly Mother’s Kitchen intervention in Andhra Pradesh. Millets remain vital in low-income and tribal diets, protecting against anaemia and undernutrition, while refined grains dominate in wealthier households. Maternal education, early marriage, and feeding practices significantly influence outcomes. The intervention markedly improved intake of protein, energy, calcium, and iron, reducing underweight. Despite awareness, taste, accessibility, and convenience, adoption is limited. Community-led, culturally relevant education is critical to sustain millet integration across generations.
